# Two-dimensional photonic crystal Bragg lasers with triangular lattice for monolithic coherent beam combining

**DOI:** 10.1038/s41598-017-10896-9

**Published:** 2017-09-06

**Authors:** Yeyu Zhu, Yunsong Zhao, Lin Zhu

**Affiliations:** Department of Electrical and Computer Engineering, Center for Optical Materials Science and Engineering Technologies, Clemson University, Clemson, SC 29634 USA

## Abstract

We demonstrate an electrically pumped, single-mode, large area, edge-emitting InGaAsP/InP two-dimensional photonic crystal (PC) Bragg laser with triangular lattice. The laser operates in the single transverse and longitudinal modes with a single lobe, near-diffraction-limited far field. We compare the performance of the triangular-lattice PC Bragg laser with the rectangular-lattice PC Bragg laser fabricated from the same wafer and find that their performances are comparable. Then, we combine two single triangular-lattice PC Bragg lasers that tilt to opposite directions by taking advantage of the symmetry of the single emitter cavity mode. The measurement results show that the combined PC Bragg lasers provide the near-diffraction-limited output beam, and the single wavelength operation is also maintained in the coherently combined broad-area PC Bragg lasers.

## Introduction

High power and single wavelength semiconductor lasers are highly desired for many applications such as sensing, ranging, high-resolution spectroscopy and interferometry. The conventional single wavelength distributed feedback (DFB) semiconductor lasers are based on the narrow stripe waveguide structure that can usually provide up to several tens of milliwatts output power^1–3^. In the narrow stripe index-guided laser diode design, the fundamental waveguide mode is used and its mode size is limited to be around several microns. However, high power operation requires a broad-area, large emitting aperture to overcome catastrophic optical damage (COD) and help with heat dissipation^4, 5^. A commercial available high-power broad-area laser diode usually has a width of ~100 *μm*. The transverse wavevector becomes comparable to the longitudinal wavevector and many waveguide modes can be supported in a broad-area laser design^6, 7^. Therefore, the single wavelength operation of a broad-area laser diode requires both the transverse and longitudinal modal control. To obtain a high power single wavelength semiconductor laser, two requirements, i.e., the large emitting aperture and simultaneous longitudinal and transverse modal control, have to be satisfied at the same time.

One candidate that can satisfy both requirements is the photonic crystal (PC) Bragg laser. The laser cavity is defined by the two-dimensional (2D) PC structure^8–10^. The PC Bragg laser is a 2D analogue of the 1D DFB laser^11, 12^. There are some interesting applications of the PC Bragg laser structure such as the 2D feedback mechanisms^13, 14^, saddle point lasing^15^, and mirrorless lasing. Furthermore, the PC Bragg laser structure with rectangular lattice has already been exploited to obtain the single-wavelength, near-diffraction-limited output beam^16–18^. The fundamental Bragg waveguide mode is selected by the periodic structure along the transverse direction and the longitudinal mode has to resonate with the periodic structure along the propagation direction. In this paper, we design the PC Bragg laser with triangular lattice for monolithic coherent beam combining (CBC) to scale up the output power. We demonstrate that the triangular-lattice PC Bragg laser and the rectangular-lattice PC Bragg laser exhibit the comparable performance, i.e., threshold current, slope efficiency, near field and far field. These results prove that the triangular-lattice PC Bragg laser also can provide the single wavelength operation with good beam quality. We further demonstrate that the coherently combined triangular-lattice PC Bragg lasers preserve the single wavelength operation and good beam quality.

For a single semiconductor laser, high power operation often results in thermooptic/optical nonlinear effects that cause deterioration in the output beam quality. To scale up the output power, CBC is often used to combine several single emitters to a laser array^19–21^. The conventional CBC approaches in chip-scale integrated systems are based on the evanescent wave coupling or leaky wave coupling which are only compatible with narrow stripe index-guided lasers or gain-guided lasers^22–26^. For monolithic CBC of broad-area lasers, we proposed and demonstrated a zigzag-like array design in our previous work to coherently combine angled-grating broad-area lasers^27^. However, since the angled grating provides mode control only in the transverse direction, the lasers usually operate with multiple longitudinal modes. In this work, we combine two single triangular-lattice PC Bragg lasers that tilt to opposite directions and use the Bragg diffraction to realize the full modal control and beam combining at the same time. Compared to the rectangular-lattice laser cavity, the laser cavity with triangular lattice is much more suitable for monolithically integrated CBC due to the geometric symmetry. The experimental results show that our approach coherently combines triangular-lattice PC Bragg lasers, maintains the single wavelength operation with the near-diffraction-limited far field, and increases the total output power.

**Figure 1:**
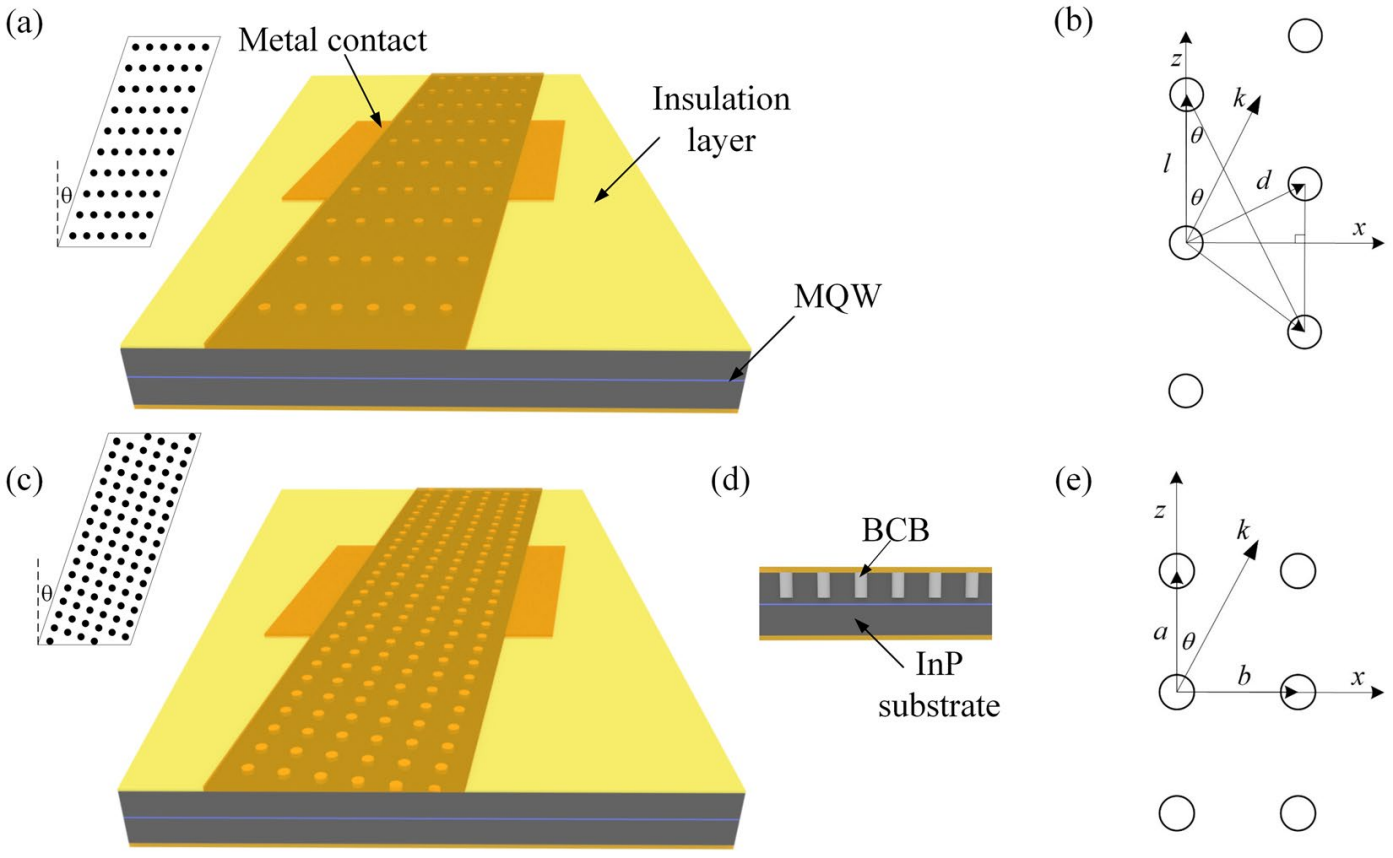
Schematic plots of the PC Bragg lasers with triangular lattice (**a**) and rectangular lattice (**c**); (**d**) Cross section of the PC Bragg lasers; Geometries and relationships with *k* vector in the triangular lattice design (**b**) and the rectangular lattice design (**e**); MQW: multiple-quantum-well; BCB: benzocyclobutene.

Figure 1 shows the schematic plots of the PC Bragg lasers. There are mainly two methods to design the PC Bragg laser cavity. One is with the rectangular lattice^17, 18^ and the other is with the triangular lattice. In the following section, the design of the PC cavities with the triangular lattice and rectangular lattice is presented to obtain the single wavelength operation. Then, in section 3 and 4, the FDTD simulation results and measurement results of the single PC Bragg lasers with different lattice designs are shown and compared. In section 5, the CBC of the PC Bragg lasers with triangular lattice is experimentally demonstrated.

## Laser design and fabrication

Figure 1(b) and (e) shows the triangular and rectangular lattice in real space, respectively. To obtain the single wavelength operation, the PC cavity needs to resonate with the lasing mode in both transverse and longitudinal directions (*k*_*x*_ and *k*_*z*_ in Fig. 1(b) and (e)). For the rectangular lattice design, we use the first order Bragg reflection in the transverse direction (*x*) and the second order Bragg reflection in the longitudinal direction (*z*). The periods of the PC cavity are indicated by *b* and *a* in the *x* and *z* directions, respectively. It is straightforward to conclude that *a* = *λ*_0_ */(n*_*eff*_ *cosθ)*, *b* = *λ*_0_ */(2n*_*eff*_ *sinθ)*. For the triangular lattice design, the effective period is *dcosθ* in the *x* direction; and along the *z* direction, the effective period is *l*. From the resonance condition, we can obtain the relation between *l* and *d*. At the same time, *l* and *d* are also geometrically related. If we use the first order Bragg reflection in both the *x* and *z* directions, the two constraints cannot be satisfied at the same time. Since the transverse mode control plays a decisive role in keeping the good beam quality^28, 29^, we use the first order Bragg reflection in the transverse direction and a high order Bragg reflection in the longitudinal direction. The integer parameter *m* is set to be the grating order number, and the derivation of *m* is shown below:

1 $${k}_{x}=ksin\theta ,\,{k}_{z}=kcos\theta $$

Along the transverse direction (*x*), we use the first order Bragg reflection:

2 $$d=\frac{{\lambda }_{0}}{{n}_{eff}sin2\theta }\,$$

Along the longitudinal direction (*z*), we use the *m*th order Bragg reflection:

3 $$l=\frac{m}{2}\frac{2\pi }{{k}_{z}}=m\frac{{\lambda }_{0}}{2{n}_{eff}cos\theta }\,$$

In addition, *l* and *d* are related geometrically in the triangular lattice by:

4 $$lsin\theta =\frac{d}{2}\,$$

By substituting Eqs (2) and (3) in Eq. (4), we can obtain the following equation:

5 $$m=\frac{1}{2si{n}^{2}\theta }\,$$

where *m* must be an integer. Equation (5) indicates that only discrete values of *θ* can be used in the triangular-lattice PC cavity design. Then we use *θ* = 10° where *m* = 16.

**Figure 2:**
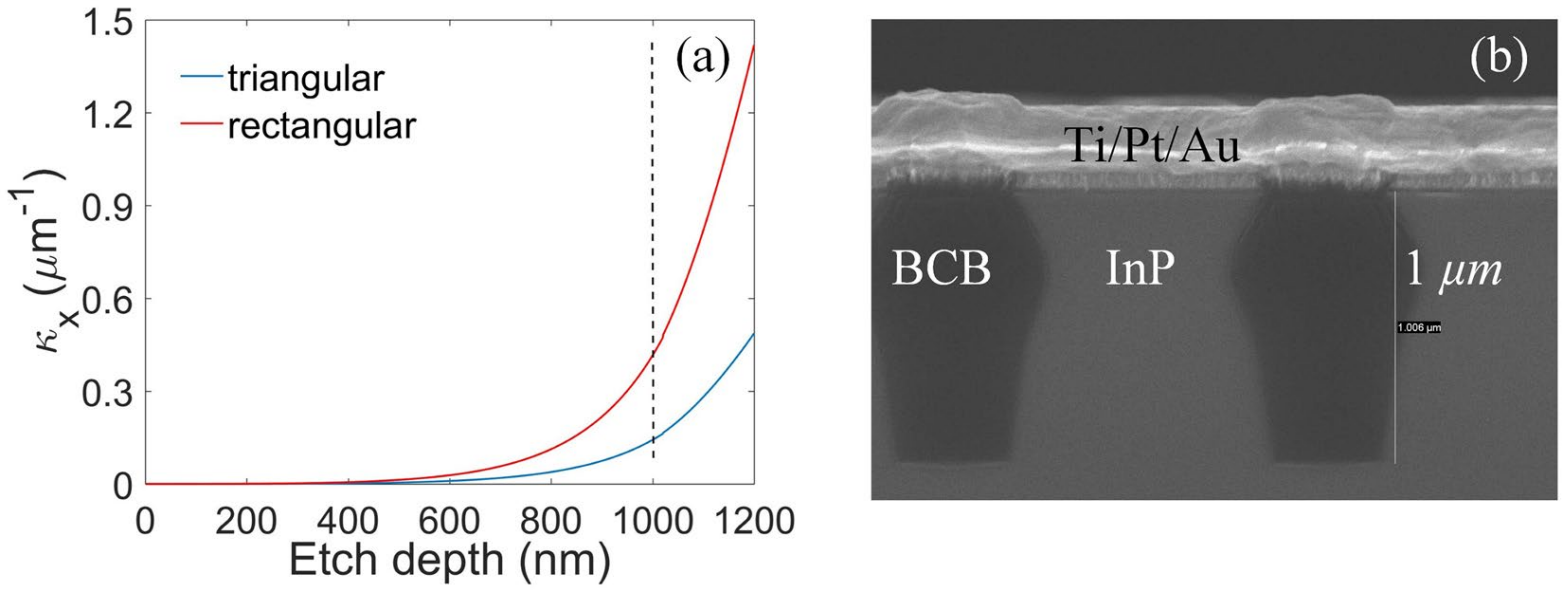
(**a**) Coupling coefficient *κ*_*x*_ of the triangular-lattice and rectangular-lattice PC cavities as a function of the etch depth; (**b**) SEM image of the cross section of the PC Bragg lasers.

The coupling coefficient along the *x* direction *κ*_*x*_ can be expressed as^30^:

6 $${\kappa }_{x}={\rm{\Gamma }}\frac{{k}_{0}^{2}}{2{k}_{x}}{\rm{\Delta }}{\varepsilon }_{1,0}\,$$

where *Δε*_1*,0*_ is the Fourier component of the dielectric perturbation, *Γ* is the confinement factor of the PC, which can be calculated from the vertical optical modal distribution in the wafer epitaxial structure. It represents how much of the optical mode interacts with the PC. Figure 2 shows the coupling coefficient *κ*_*x*_ with respect to the etching depth (the radius of the hole is set to be 350 *nm*). The *κ*_*x*_ is selected to be ~*0.1*/*μm*, which is similar to the design of angled grating broad-area lasers. Therefore, we will set the etching depth around 1 *μm* without etching the quantum wells region, as shown in Fig. 2. For our practical design, the laser has a width *W* of 100 *μm* and a length *L* of 1.3 *mm*. The products of the coupling coefficients and the device dimensions, *κ*_*x*_ *W* and *κ*_*z*_ *L*, are important for the PC design. If *κ*_*x*_ *W* and *κ*_*z*_ *L* are too small, the confinement in the transverse and longitudinal directions is not strong enough to provide the mode selection. If *κ*_*x*_ *W* and *κ*_*z*_ *L* are too large, high order spatial modes can lase and spatial hole burning can occur. The detailed discussion about the relation between the coupling coefficients and the device dimensions is given in ref. 30. The III–V layers are etched through using plasma etching with HBr gas which can attack all the III–V layers at a similar rate. However, the undercut profile^31^ might be obtained as shown in Fig. 2(b). The etching profile can be improved by balancing the chemical etching and the physical etching in the inductively coupled plasma system (ICP). The etching depth of the two types of the laser cavities is set to be identical. 100 periods in the transverse direction are chosen resulting in a total width of ~140 *μm*. The PC Bragg lasers are fabricated in an InP-based multiple-quantum-well (MQW) epitaxy wafer. The details of the epitaxy wafer are given in ref. 27. The MQW layer is tensile strained, so the transverse electric (TE) lasing mode is preferred. The fabrication process consists of a series of steps of lithography, etching, planarization and metallization, which is the same as the process flow described in ref. 27. The laser chip is cleaved to the length of ~1.3 *mm*, and then is bonded on a c-mount for measurement. The laser diode is placed inside a cryostat to obtain the low temperature operation (~210 *K*). During fabrication, the semiconductor materials are etched and replaced by the insulation material (BCB), which results in the higher electrical resistance. Besides, the etched surface may introduce some surface defect states and increase the non-radiative recombination rate. At the low temperature, semiconductor materials have higher optical gain due to the reduced rate of non-radiative, thermal transitions. The intrinsic loss of the laser diode can be reduced. Thus, it is easier to obtain the electrically pumped continuous wave (CW) operation with a relatively low lasing threshold at the low temperature. The detailed description of measurement setups for characterizing laser diodes can be found in ref. 30.

## FDTD simulation

**Figure 3:**
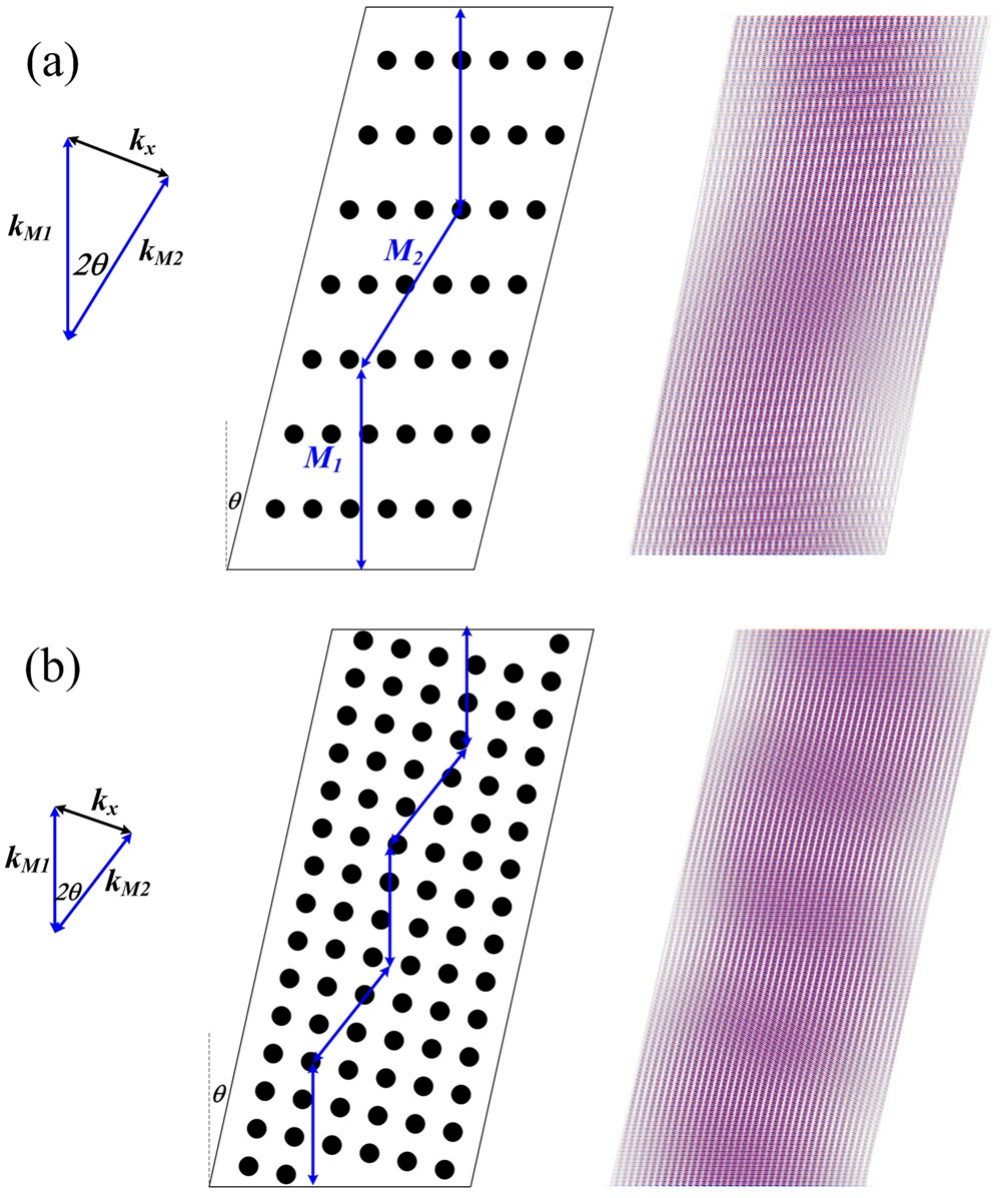
Wave coupling and cavity modes in the triangular-lattice PC cavity (**a**); the rectangular-lattice PC cavity (**b**).

The cavity modes in the PC cavity with the rectangular and triangular lattice can be obtained by solving the 2D coupled wave equations^30^. The calculated modes in the triangular-lattice cavity and the rectangular-lattice cavity, consist of two plane-wave like components that resonate with the PC cavity, *M*_*1*_ and *M*_2_, as shown in Fig. 3 ^27^. The angles between *M*_1_ */M*_2_ and the PC cavity direction are both equal to *θ*, the cavity tilt angle. The wavevectors of *M*_1_, *M*_2_ and the PC cavity satisfy the resonance condition: *k*_*M*1_ + *k*_*M*2_ = *k*_*x*_, as shown in Fig. 3. The propagation direction of the *M*_1_ component is perpendicular to the facet and that of *M*_2_ is tilted. When *M*_1_ is reflected by the facet, it will be fed back to the cavity; but for *M*_2_, it will be lost. Therefore, once used as the laser cavity, the PC cavity will self-adaptively select the cavity mode with the maximum *M*_*1*_ and the minimum *M*_*2*_ component at the facets. The cavity mode profiles for both designs are obtained by using the FDTD method, as shown in Fig. 3. Due to the large modal discrimination and strong spatial filtering provided by the angled cavity design^32, 33^, the broad-area (>100 *μm*) PC Bragg lasers can provide the stable single wavelength operation. From Fig. 3, it is found that the coupling strength of the triangular-lattice PC cavity is weaker than the rectangular-lattice PC cavity, which agrees with the calculations in section 2.

## Experimental results

### Single PC Bragg laser

**Figure 4:**
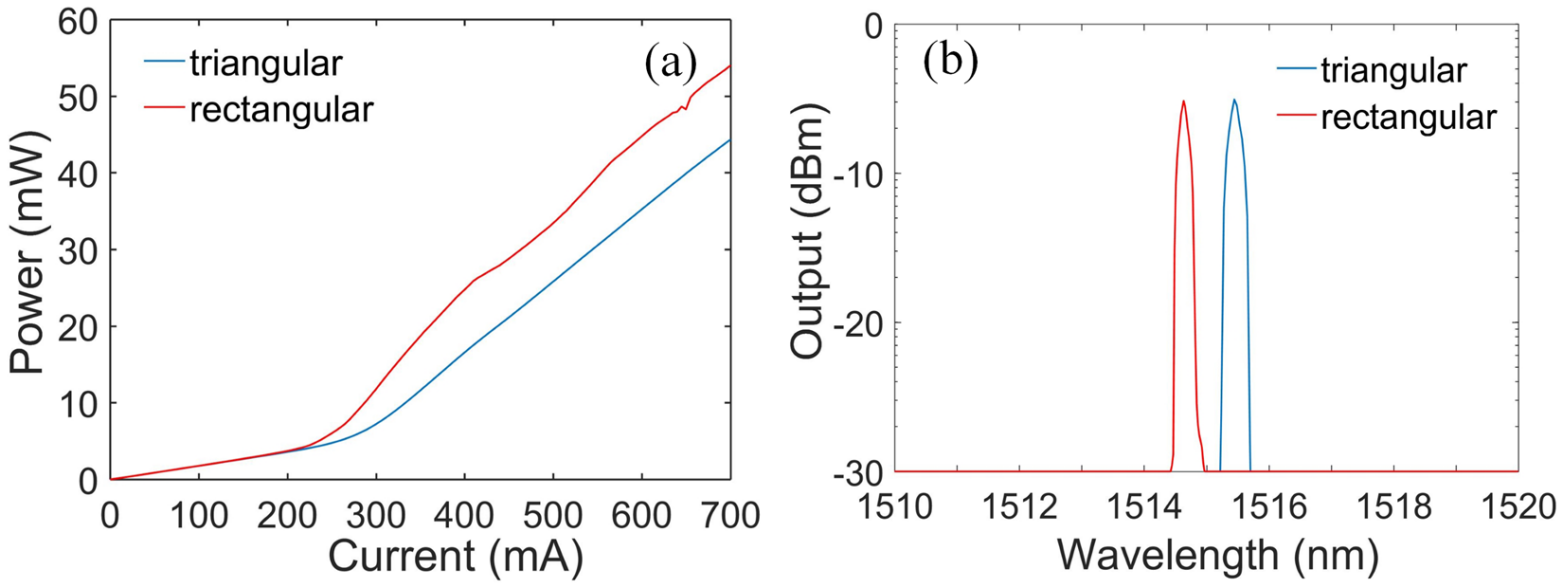
LI curves (**a**) and optical spectra (**b**) of the single triangular-lattice and rectangular-lattice PC Bragg lasers with the same parameters (the pump current is set to be 700 mA for the optical spectra).

We take the measurement of LI curves, optical spectra, “near field” and far field of the single PC Bragg lasers. The measurement results are obtained in a cryostat system, and the temperature is set at 210 *K*. Figure 4 shows the LI curves and optical spectra of the triangular-lattice and rectangular-lattice PC Bragg lasers. The CW current source is used as the pump source. In Fig. 4, we include the LI curve and optical spectrum of the rectangular-lattice PC Bragg laser for comparison. The threshold current of the triangular-lattice (the rectangular-lattice) PC Bragg laser is ~240 *mA* (~200 *mA*) and the slope efficiency is ~0.14 *W*/*A* (~0.15 *W/A*). It is obvious that the threshold current and slope efficiency of the triangular-lattice PC Bragg laser are comparable to the rectangular-lattice PC Bragg laser. The threshold current of the triangular-lattice PC Bragg laser is a little bit higher due to the relatively weaker coupling strength. In the angled-grating broad-area laser^27^, since the angled grating provides the distributed feedback in the transverse direction, the longitudinal modes are determined by the two end-facets. The angled-grating broad-area laser usually operates with multiple longitudinal modes. However, in the PC Bragg laser, because both the transverse and longitudinal wavevectors are resonant with the PC, the PC Bragg laser is able to operate with the single wavelength. The optical spectra shown in Fig. 4(b) prove that the PC Bragg lasers with triangular lattice and rectangular lattice both can provide the stable single wavelength operation at around 1515 *nm* with a sidemode suppression ratio exceeding ~22 *dB*. These results suggest that the longitudinal mode is well controlled by the PC cavity.

**Figure 5:**
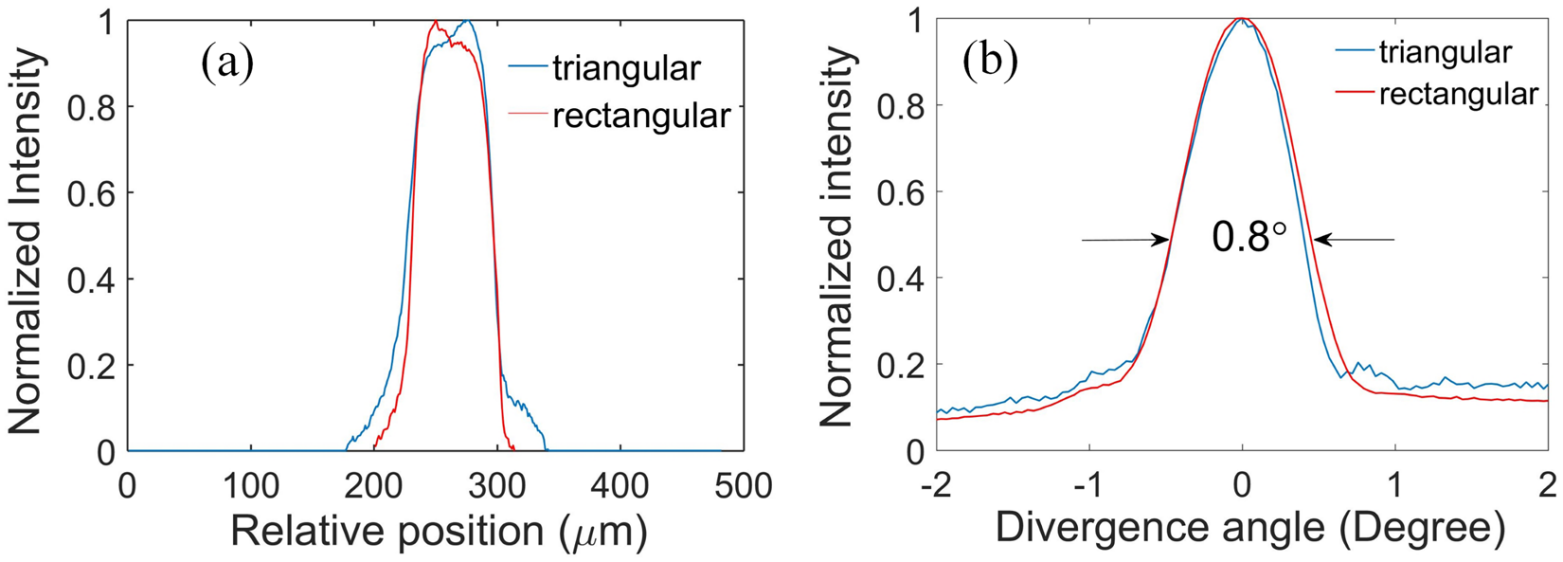
“Near field” profiles (**a**) and far field profiles (**b**) of the single triangular-lattice and rectangular-lattice PC Bragg lasers with the same parameters.

Figure 5(a) and (b) show the “near field” profiles and far field profiles of the single triangular-lattice and rectangular-lattice PC Bragg lasers, respectively. It should be pointed out that the “near field” profile obtained in this paper refers to the emission pattern of the laser at the output edge facet captured by an infrared (IR) camera with an objective lens. As shown in Fig. 5, the single triangular-lattice and rectangular-lattice PC Bragg lasers have almost the same “near field” profiles and far field profiles. The “near field” profile shows that the width of the emitting aperture is around 115.4*μm*. The far field profile shows a single lobe with the divergent angle around 0.8°, indicating the near-diffraction-limited beam quality. The numerical criterion of the near-diffraction-limited beam quality of a broad area diode laser can be found in ref. 7. From these results, though the coupling strength of the triangular-lattice PC is weaker, we find that the performance of the PC Bragg laser with triangular lattice is comparable to the rectangular-lattice PC Bragg laser. We have demonstrated that the single wavelength laser with the near-diffraction-limited output beam can be obtained by using the triangular-lattice PC cavity design. A unique advantage associated with the triangular-lattice PC cavities is that they can be coherently combined without any external components by use of the symmetry of the geometry and cavity mode.

### Coherently combined PC Bragg lasers

**Figure 6:**
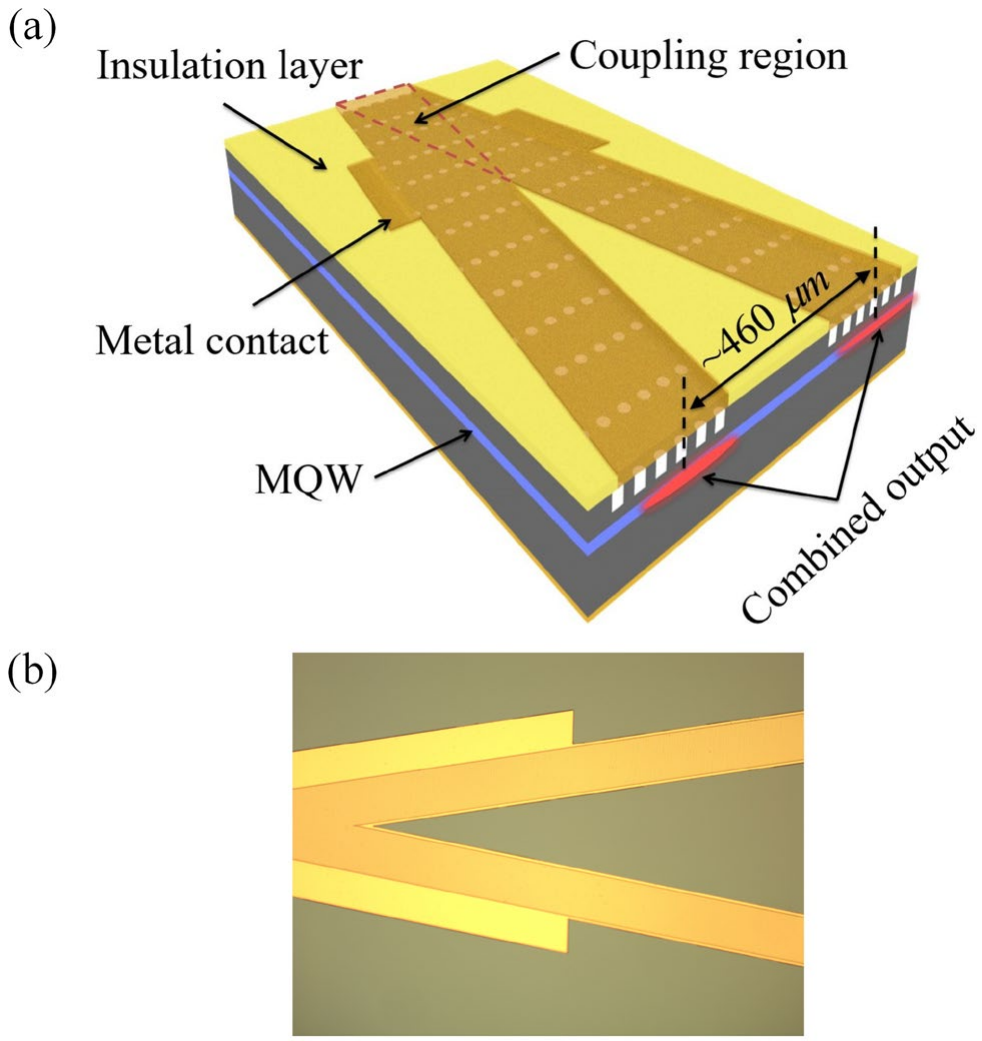
Schematic plot (**a**) and microscopic image (**b**) of the coherently combined PC Bragg lasers.

In this section, to implement the chip-scale monolithic coherent beam combining of the PC Bragg lasers, two single emitters are combined. Figure 6 shows the schematic plot and the microscopic image of the combined PC Bragg lasers. Two PC Bragg lasers with triangular lattice tilting to the opposite directions overlap at one facet and the overlapped area defines the beam combining/coupling region. Unlike the conventional PC Bragg laser^17, 18^, the triangular lattice is used instead of the rectangular lattice in this work because the triangular lattice has the same symmetry as the combined lasers design and leads to the complete lattice overlap of the two single emitters at the coupling region.

**Figure 7:**
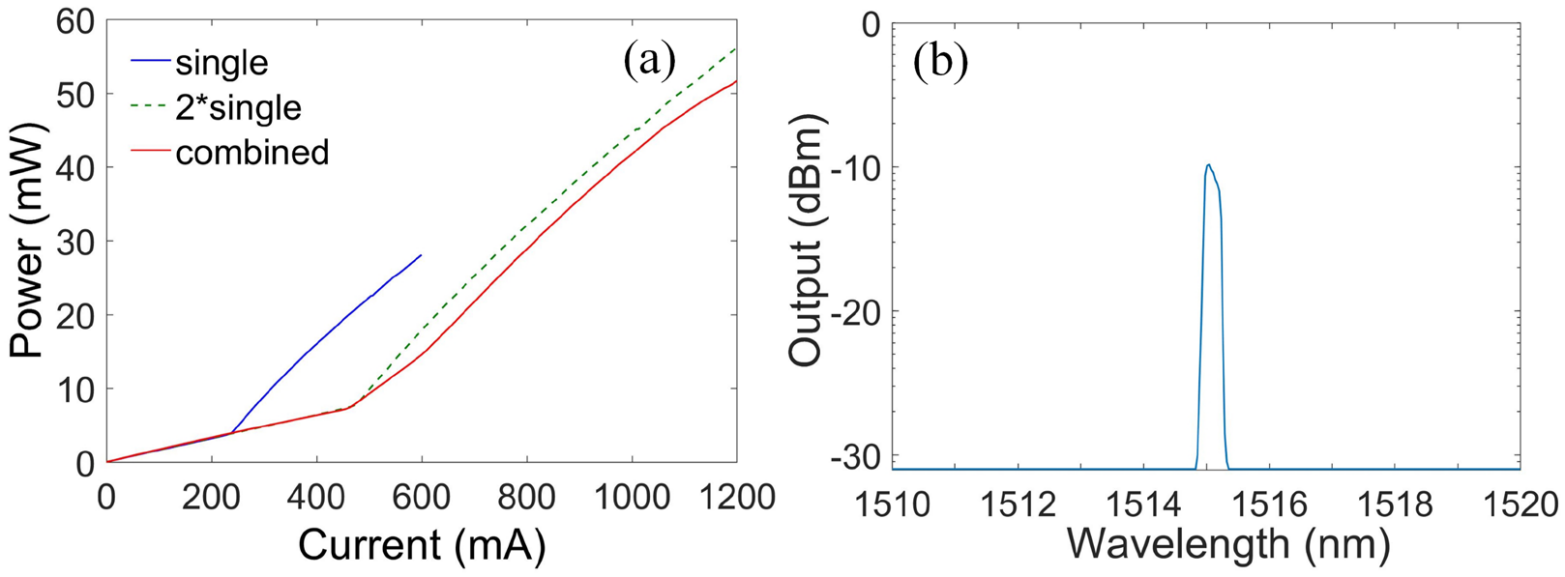
Measurement results of the LI curves and the optical spectrum. (**a**) L-I curves of the combined PC Bragg lasers (red solid line), the single PC Bragg laser (blue solid line) and the two single PC Bragg lasers (green dashed line); (**b**) Optical spectrum of the combined PC Bragg lasers (the pump current is set to be 1,000 mA).

Figure 7 shows the LI curve and the optical spectrum of the combined PC Bragg lasers with triangular lattice. The threshold current is around 400 *mA* and the slope efficiency is about 0.14 *W*/*A*. In Fig. 7(a), we also show the LI curves of the single PC Bragg laser and the twice output power of the single PC Bragg laser in the same figure for comparison. As for the ideal power combining efficiency, the output power of the coupled emitters should be twice the output power of a single emitter when the injected current is doubled. From Fig. 7(a), the combining efficiency of the combined PC Bragg lasers is around 90%. In our experiments, more than twenty samples are measured, and the combining efficiency is 88% ± 3%. The imperfect power combining efficiency is mainly caused by uneven power distribution of different emitters induced by the non-uniformity of the fabrication, which can be improved through the optimized dry etching, planarization and metallization processes. In Fig. 7(b), the optical spectrum shows the stable single wavelength operation. The pump current is set at 1,000 mA.

**Figure 8:**
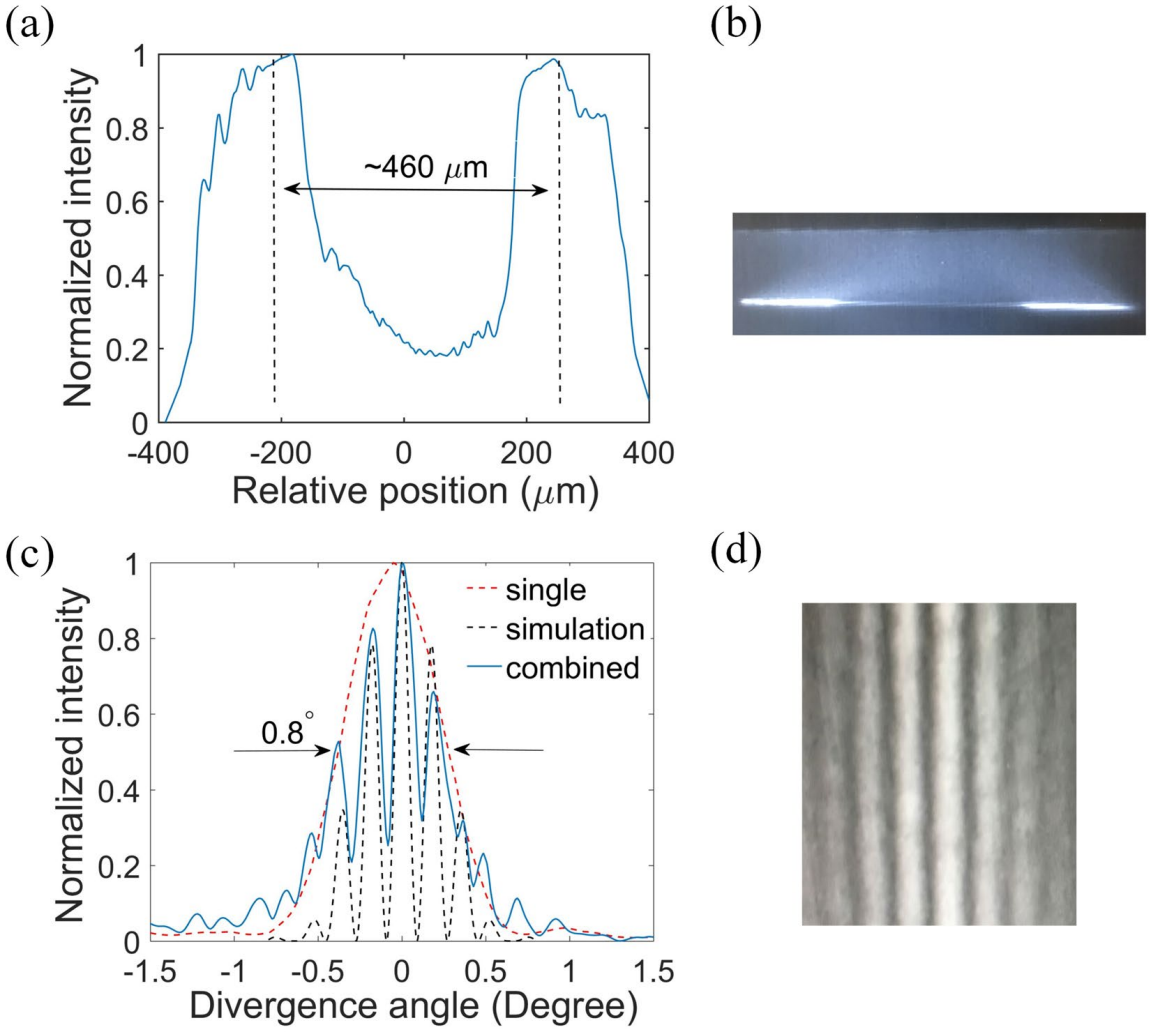
The “near field” profile (**a**) and IR image (**b**) of the combined PC Bragg lasers; (**c**) The far field profiles of the uncoupled single PC Bragg laser, the combined PC Bragg lasers and the simulation result; (**d**) The far field IR image of the coherently combined PC Bragg lasers.

Figure 8(a) and (b) shows the “near field” profile and IR image of the combined PC Bragg lasers. The distance between the two emitting regions (~460*μm*) and the width of each emitting region (~110*μm*) indicate that the light indeed emits from the designed emitters, which is also shown in the schematic plot Fig. 6(a). The far field profile and IR image of the combined PC Bragg lasers is shown in Fig. 8(c) and (d). We also compare the far field profile of the combined emitters with that of the uncoupled single emitter. The uncoupled single emitter was fabricated on the same wafer with the combined emitters. The design parameters are the same. If the coupled emitters are coherently combined and in-phase, they will constructively interfere in the far field and the overall envelop of the interfered far field remains the same as that of the single emitter. The only difference is that there would be interference patterns present within the overall envelop for the combined emitters^27^. As shown in Fig. 8(c) and (d), our measurement results show the expected far field profile. It is clear that the overall far field envelop of the combined PC Bragg lasers is similar to that of the uncoupled single PC Bragg laser. The fine interference patterns in Fig. 8(c) and (d) prove that the two emitters are coherently combined. The overall FWHM divergence angles (~0.8°) are much smaller than a conventional broad-area laser (∼ 10°)^28, 29^. We extract the distance between the two emitters and the width of each emitter from the measured “near field” profile in Fig. 8(a). We assume that the two emitters are in-phase and calculate the far field pattern by using the standard diffraction theory. The calculation result is shown in Fig. 8(c) in the grey dashed line and agrees well with the measured result. The small difference in the divergence angles between the single emitter and the combined emitters is mainly due to the different “near field” distribution induced by the non-uniformity of fabrication and current injection^27^.

In the previous work, the similar zigzag array design has been used to coherently combine angled-grating broad-area lasers^27^. Multiple longitudinal modes appear in the angled-grating broad-area lasers. Here, we replace the 1D grating with the 2D triangular-lattice PC cavity. Since the PC cavity is able to provide the mode control in both the transverse direction and longitudinal direction, the single wavelength operation can be obtained. With the triangular lattice design, the coupling region shares the same lattice structure as the single emitter and there is no interface mismatch between the combined emitters, which lead to more uniform etching profile. Therefore, we can realize the monolithic coherent beam combining and the single wavelength operation at the same time in the combined broad-area PC Bragg laser structures. The scalability of this zigzag type of combination is analyzed and compared to the tree like structure and other common cavities in ref. 34. Our results are important to realize the high power, single wavelength semiconductor laser sources on a single chip by using the coherent beam combining of PC Bragg laser structures.

## Conclusion

We demonstrate the triangular-lattice PC Bragg laser operating at the single wavelength for monolithically integrated coherent beam combining. From the measurement results, we show that the performance of the triangular-lattice PC Bragg laser is comparable to that of the rectangular-lattice PC Bragg laser. Furthermore, we demonstrate the coherent beam combining of two PC Bragg lasers with the triangular lattice. Our experimental results show that the combined broad-area PC Bragg lasers provide the near-diffraction-limited output beam and the single wavelength operation at the same time.
